# Aβ Oligomer Elimination Restores Cognition in Transgenic Alzheimer’s Mice with Full-blown Pathology

**DOI:** 10.1007/s12035-018-1209-3

**Published:** 2018-07-12

**Authors:** Sarah Schemmert, Elena Schartmann, Christian Zafiu, Bettina Kass, Sonja Hartwig, Stefan Lehr, Oliver Bannach, Karl-Josef Langen, Nadim Joni Shah, Janine Kutzsche, Antje Willuweit, Dieter Willbold

**Affiliations:** 1 Institute of Complex Systems, Structural Biochemistry (ICS-6), Forschungszentrum Jülich, 52425 Jülich, Germany; 2 Institute of Clinical Biochemistry and Pathobiochemistry, German Diabetes Center at the Heinrich Heine University Düsseldorf, Leibniz Centre for Diabetes Research, Düsseldorf, Germany; 3 German Center for Diabetes Research (DZD), Partner Düsseldorf, Germany; 4 Institute of Neuroscience and Medicine, Medical Imaging Physics (INM-4), Forschungszentrum Jülich, 52425 Jülich, Germany; 5 Clinic for Nuclear Medicine, RWTH Aachen University, Aachen, Germany; 6 Department of Neurology, Faculty of Medicine, JARA, RWTH Aachen University, Aachen, Germany; 7 Institut für Physikalische Biologie, Heinrich-Heine-Universität Düsseldorf, Düsseldorf, Germany

**Keywords:** Alzheimer’s disease therapy, Amyloid-β oligomer elimination, Transgenic mice, d-enantiomeric peptides, Target engagement

## Abstract

Oligomers of the amyloid-β (Aβ) protein are suspected to be responsible for the development and progression of Alzheimer’s disease. Thus, the development of compounds that are able to eliminate already formed toxic Aβ oligomers is very desirable. Here, we describe the in vivo efficacy of the compound RD2, which was developed to directly and specifically eliminate toxic Aβ oligomers. In a truly therapeutic, rather than a preventive study, oral treatment with RD2 was able to reverse cognitive deficits and significantly reduce Aβ pathology in old-aged transgenic Alzheimer’s Disease mice with full-blown pathology and behavioral deficits. For the first time, we demonstrate the in vivo target engagement of RD2 by showing a significant reduction of Aβ oligomers in the brains of RD2-treated mice compared to placebo-treated mice. The correlation of Aβ elimination in vivo and the reversal of cognitive deficits in old-aged transgenic mice support the hypothesis that Aβ oligomers are relevant not only for disease development and progression, but also offer a promising target for the causal treatment of Alzheimer’s disease.

## Introduction

The most common form of dementia worldwide is Alzheimer’s disease (AD), a progressive and incurable neurodegenerative disease [1–3]. Current treatments with acetylcholinesterase inhibitors and an NMDA receptor antagonist are only able to treat some symptoms and hold the risk of unpleasant side effects [4]. Thus, there is an urgent need for the development of new therapeutic strategies for the causal treatment of AD [5].

The pathological hallmarks of AD are neurofibrillary tangles, consisting of hyperphosphorylated tau protein, progressive neurodegeneration, and the accumulation of toxic amyloid-β (Aβ) species [1, 5]. The central dogma for the development of AD is the so-called amyloid cascade hypothesis [6, 7]. It states that an imbalance between the production and clearance of Aβ in the brain of affected people is responsible for neurodegeneration and finally dementia. Monomeric Aβ, a cleavage product of the proteolytic processing of the amyloid protein precursor (APP) by a β- and a γ-secretase, aggregates into Aβ oligomers and finally into amyloid fibrils, which are found in AD plaques and were previously considered to be the cause of cognitive deficits [8]. Nowadays, more and more evidence exists which suggests that instead of Aβ monomers or fibrils, small and diffusible Aβ oligomers may be the main cause for the development and progression of cognitive decline in AD [1, 9]. Therefore, their elimination is highly desirable for therapy development and some attempts pertaining to this have been made in the past [10, 11]. However, to date, no treatment strategy based on the prevention of Aβ oligomer formation with small molecule inhibitors against β- and γ-secretases has successfully completed clinical phase III. Many developments have been discontinued because of missing therapeutic benefits and side effects. In addition, therapeutic antibodies directed against monomeric or fibrillary Aβ, or both, have so far shown no therapeutic efficacy in clinical phase III trials either, and have been associated with serious side effects called amyloid-related imaging abnormalities (ARIA), which seem to be a consequence of an antibody-related activation of the immune system. Recent passive immunization approaches have tried to target Aβ oligomers more specifically by using stabilized oligomers of various sizes or conformations as antigens. However, their efficacy still needs to be demonstrated in clinical trials [12].

We propose a treatment strategy directed towards the specific and direct elimination of toxic Aβ oligomers, irrespective of their size and conformation, via their direct disruption, rather than by relying on the immune system for their destruction. This is achieved by binding our drug candidate to monomeric Aβ, stabilizing it in an aggregation-incompetent state, and thereby shifting the chemical equilibrium away from toxic Aβ oligomers [13]. By using this approach, it is both possible to prevent the formation of oligomers and to disrupt already formed Aβ oligomers. Successful proof of this mode of action has been achieved for the orally available compound D3 and its derivatives in vitro by demonstrating the removal of preformed, toxic Aβ oligomers [14]. D3 has been identified by mirror image phage display and is a peptide that consists solely of d-enantiomeric amino acid residues. In vivo D3 has been shown to improve pathology and cognition after administration via different routes, including oral application [14–19]. Additionally, pharmacokinetic studies have demonstrated the proteolytic stability and bioavailability of D3 [20, 21]. The compound investigated in this study, RD2, is a derivative of D3, with a rationally repositioned amino acid sequence to enhance Aβ oligomer elimination efficiency. Previously, with the use of the Aβ-QIAD (quantitative determination of interference with Aβ aggregate size distribution) assay, we were able to demonstrate that RD2 is more effective for the elimination of toxic Aβ oligomers in vitro [14, 22]. This assay is based on the fractionation of Aβ_(1–42)_-assemblies (Aβ monomers, oligomers, protofibrils, larger aggregates) according to their sizes (after a predefined incubation time) by density gradient ultracentrifugation (DGC). Using this assay, it is possible to quantitatively determine the potency of a compound to reduce preformed toxic Aβ oligomers in vitro. According to Brener et al., the toxicity of oligomeric Aβ species is reduced after co-incubation with Aβ oligomer eliminating compounds [14]. Besides oligomer removal, several tests illustrate the in vitro efficacy of RD2 to reduce Aβ_(1–42)_ fibril formation, the catalytic effect of preformed seeds on Aβ_(1–42)_ aggregation, and to reduce the Aβ_(1–42)_-mediated cell toxicity [22]. Two preclinical proofs of concept studies have already demonstrated the efficacy of RD2 by improving cognition in two different transgenic AD mouse models [22, 23].

In the present study, we set out to support the suggested mode of action by demonstrating in vivo target engagement, which would be the reduction of the Aβ oligomer concentrations in the brains of RD2-treated mice, as compared to placebo-treated mice. This, however, requires an appropriate detection and quantitation method for Aβ oligomers. One major challenge associated with this is the ability to clearly distinguish between monomeric and oligomeric/aggregated Aβ species. To adequately meet this need, some attempts have been made, mostly based on ELISA techniques [24, 25], and include a method known as the sFIDA (surface-based fluorescence intensity distribution analysis) assay, which has been developed to quantify Aβ oligomers. The sFIDA assay uses a sandwich-ELISA biochemical setup using anti-Aβ antibodies to capture all Aβ species to a glass surface. Fluorescence-labeled anti-Aβ antibodies act as probes that recognize the same, or overlapping, Aβ epitopes as the capture does. This avoids any contribution of Aβ monomers to the measurement. In contrast to conventional ELISAs, however, the readout is obtained by taking the fluorescence micrographs from the glass surface using total internal reflection (TIRF) microscopy, which then yields single-particle sensitivity. The absolute Aβ oligomer concentrations are then calculated on the basis of a suitable calibration standard [26–31].

Besides the demonstration of in vivo target engagement, the aim of the current study was to proof the proposed mode of action, i.e., the elimination of already formed Aβ oligomers, in vivo in a therapeutic, rather than preventive, setting. Therefore, we challenged the efficacy of the drug candidate RD2 by using it for the oral treatment of old-aged APP/PS1 transgenic AD mice exhibiting full-blown pathology, severe cognition impairments, and behavior deficits before the start of treatment.

## Material and Methods

### Mice

In the present study, the double transgenic APPswe/PS1ΔE9 (APP/PS1) AD mouse model, introduced by Jankowsky et al. in 2004 [32], was used, its pathology and behavioral deficits having been well characterized. At 6 months of age, the mice develop Aβ deposits, gliosis, and cognitive deficits, which are especially detectable in the Morris water maze (MWM). The pathology and cognitive deficits progressively intensify with age [32–35]. APP/PS1 mice were ordered from the Jackson Laboratory (Jackson Laboratory, USA) and bred in-house, in a controlled environment, on a light/dark cycle (12/12 h), with 54% humidity and a temperature of 22 °C. Food and water were available ad libitum. All experiments were performed in accordance with the German Law on the protection of animals (TierSchG §§ 7–9) and were approved by a local ethics committee (LANUV, North-Rhine-Westphalia, Germany, reference number: 84-02.04.2011.A359).

Aged male APP/PS1 mice (*n* = 21) and their non-transgenic littermates (*n* = 11) were tested in the present study (average age at treatment initiation: 18 months ± 3 weeks). During the study, all mice were housed single caged.

### Peptide

RD2 was purchased from CBL Patras (CBL Patras, Greece) and its amino acid residue sequence is as follows: H-ptlhthnrrrrr-NH_2_.

### Treatment

Mice were treated orally, every day, for 12 weeks with either RD2 (*n* = 11) or placebo (drinking water) (*n* = 10) both formulated in tailor-made jellies composed of gelatin (30% sucrose, 10% sucralose, 18.75% instant gelatin) (Dr. Oetker, Bielefeld, Germany). The mice ate each single jelly completely and voluntarily. Every week, the RD2 amount in the jellies was adjusted to the average body weights of the mice to achieve a daily dose as close as possible to 200 mg/kg. During the final weeks of treatment, for example, each jelly for the RD2 treatment group contained 6.7 mg RD2.

### Behavioral Assessments

In all experiments conducted, the behavioral performance of RD2- and placebo-treated mice was compared to those of non-transgenic littermates, which were left untreated and assured for the quality of the behavioral assessments.

#### Nesting Behavior and Marble Burying

To assess species-typical behavior, nesting behavior and marble burying were performed. Both protocols were adopted from Deacon [36, 37]. In short, for nesting behavior, mice were placed in a new cage with a fresh nestlet (Sniff, Germany) 1 h before the dark phase of the animal house. The next morning, the built nests were scored according to Deacon’s scores from 0 to 5, whereby 0 represents no nest and 5 represents a perfect nest [36]. For marble burying, mice were placed in a new cage with 5-cm-deep wood chip bedding on which 12 glass marbles (diameter: 1.6 cm, weight: 5.3 g) were laid down in a predefined order. After 30 min, the number of marbles buried was counted.

#### Open Field Test

The open field test is an experimental arrangement for the quantitative representation of the explorative and anxiety behavior of animals [38]. After 30 min of habituation to the experiment’s room, mice were placed in a square-shaped arena (45 cm × 45 cm × 45 cm). The arena was imaginarily divided into two zones: center and border zone (center: 19 cm × 19 cm, border: space around the center zone). Mice were allowed to observe the arena for 30 min. They were recorded with a camera-driven tracking system, Ethovision 11 (Noldus, Wageningen, the Netherlands). For analysis, the first 25 min of the recording was subdivided into five time slots (1: 0–5 min; 2: 5–10 min; 3: 10–15 min; 4: 15–20 min; 5: 20–25 min). The duration of stay in the center and the border zone was evaluated concerning explorative and anxiety behavior, both in total and separately for each time slot.

#### Morris Water Maze

The MWM is a spatial learning test to investigate cognitive impairments. The apparatus used consists of a circular white pool (120 cm in diameter and 60 cm in height), filled with water (24 ± 1 °C) to a depth of 30 cm. The water is rendered opaque with the addition of a non-toxic white pigment. The pool is imaginarily subdivided into four quadrants: northeast (NE, target quadrant with an invisible round platform 1 cm below the surface), southeast (SE), southwest (SW), and northwest (NW). The protocol was modified according to Morris et al. [39]. During the training, the mice swam four 60-s trials daily, starting from different quadrants on five consecutive days. If they did not find the hidden but fixed platform within the 60 s of a trial, they were set on the platform for 10 s to orient themselves before they were returned to their cages. Between each trial, the mice had a recovery period of 60 s under a heating lamp to avoid a decrease in body temperature. On the sixth day, the probe trial was performed, in which the mice had to swim in the pool for 60 s without a platform. During the trials, the mice were tracked with Ethovision 11 (Noldus, Wageningen, the Netherlands). The following parameters were analyzed for the training days: escape latency to platform (s), covered distance (cm), swimming speed (cm/s), and the duration in the platform quadrant (s). The duration in the platform quadrant was also analyzed for the probe trial.

### Plasma and Tissue Collection

Mice were anesthetized with 100 mg/kg ketamine (bela-pharm, Vechta, Germany) and 0.3 mg/kg medetomidine (Dormilan, alfavet, Neumünster, Germany) intraperitoneally before the final collection of blood samples by terminal cardiac puncture. Brains were removed, divided into the two hemispheres and snap frozen in − 80 °C isopentane. The left hemispheres were used for immunohistochemistry (IHC); the right hemispheres were used for biochemical analysis.

### Immunohistochemistry and Biochemical Analysis

#### Immunohistochemistry

IHC was performed on 20-μm sagittal frozen brain sections. In brief, room tempered sections were fixed in 4% paraformaldehyde (10 min, room temperature). For antigen retrieval, sections were incubated in 70% formic acid (5 min, room temperature). Elimination of endogenous peroxidases was performed with 3% H_2_O_2_ in methanol (15 min, room temperature). In between, the sections were washed three times for 5 min in 1% Triton in TBS (TBST). Sections were incubated with the primary antibody over night at 4 °C in a humid chamber (6E10: 1:2500, Bio Legend, San Diego, USA; GFAP: 1:1000, DAKO, Agilent Technologies, Santa Clara, USA; CD11b: 1:2500, Abcam, Cambridge, UK) in TBST with 1% bovine serum albumin (BSA), followed by incubation with a biotinylated secondary anti-mouse or anti-rabbit antibody (both 1:1000 in TBST + 1% BSA, Sigma-Aldrich, Darmstadt, Germany). Staining was visualized with the use of 3,3′-diaminobenzidine (DAB) enhanced with saturated nickel ammonium sulfate solution. Sections were mounted with DPX Mountant (Sigma-Aldrich, Darmstadt, Germany) after washing in an ascending alcohol series.

To avoid differences in staining intensity and light exposure, which might affect measurements, all slides were stained in one batch and were acquired in one microscopy session. Sections were visualized with the use of a Zeiss SteREO Lumar V12 microscope and the according software (Zeiss AxioVision 6.4 RE) or a Leica LMD6000 microscope and the according software (LAS 4.0 software). Quantification was performed with ImageJ (National Institute of Health, Bethesda, USA). The plaque count of RD2- (*n* = 11) and placebo-treated (*n* = 8) mice was analyzed in the cerebrum (8–10 slides/mouse), cortex (5–6 slides/mouse), and hippocampus (8–10 slides/mouse). The astrogliosis of RD2- (*n* = 8) and placebo-treated (*n* = 7) mice was analyzed in the cortex (6 slides/mouse with 6 equally distributed pictures per slide) and the hippocampus (6 slides/mouse). The activated microglia of RD2- (*n* = 9) and placebo-treated (*n* = 9) mice were analyzed in the cortex (7 slides/mouse) and the hippocampus (7 slides/mouse).

#### Aβ ELISA

For the generation of three fractions (Tris, diethanolamine (DEA), and formic acid (FA) fractions), the right hemispheres of RD2- (*n* = 10) and placebo-treated (*n* = 8) mice were used. To obtain the Tris fraction, hemispheres were homogenized 2 × 20 s at 6500 rpm (Precellys® 24, Bertin Instruments, Montigny-le-Bretonneux, France) with Tris buffer (pH 8.3, 20 mM Tris, 250 mM NaCl, protease and phosphatase inhibitors (both Roche, Basel, Switzerland)). Afterwards, the homogenized samples were sonicated (5 min) and centrifuged (30 min, 175,000×*g*, 4 °C). Supernatant was taken as the Tris-soluble fraction. DEA fractions were gained after dissolving the pellet in 2% DEA, incubation (1 h on ice), and centrifugation (30 min, 175,000×*g*, 4 °C). Supernatant was taken as the DEA fraction. The pellet was dissolved in 70% FA, incubated (1 h on ice), and centrifuged (30 min, 175,000×*g*, 4 °C). Supernatant was taken as the FA fraction. All fractions were snap frozen in liquid nitrogen and stored at − 80 °C.

Aβ x–40 and Aβ x–42 ELISAs were purchased from BioLegend (BioLegend, San Diego, USA) and performed according to the manufacturer’s protocol with the three brain homogenate fractions described above. All samples were measured as duplicates.

### Surface-Based Fluorescence Intensity Distribution Analysis Assay

Surface-based fluorescence intensity distribution analysis (sFIDA) assays were performed in 384 flat-bottom square well microplates (Sensoplate Plus, Greiner Bio-One GmbH, Frickenhausen, Germany) with a glass bottom of 170 μm thickness. The glass bottom of the microplate was silanized with APTES (99%; 3-aminopropyltriethoxysilane; Sigma-Aldrich, Germany) by vapor deposition. For this procedure, a microplate was placed in a desiccator above a solution of 5% APTES in toluene (99% Sigma-Aldrich, Germany), in an argon atmosphere for 1 h before removing the APTES solution and drying for 2 h in a vacuum. Two-millimolar succinimidyl carbonate-poly-(ethylene glycol)-carboxymethyl (MW 3400, Laysan Bio, Arab, USA) in ddH_2_O was added to the wells, incubated for 4 h, and washed three times after incubation. This procedure covalently links PEG to APTES and presents carboxylic acids to the surface, which are activated by 200 mM *N*-(3-dimethylaminopropyl)-*N*′-ethylcarbodiimide hydrochloride (98%; Sigma-Aldrich, Germany) and 50 mM *N*-hydroxysuccinimide (98%; Sigma-Aldrich, Germany), and incubated for 30 min. After washing three times with ddH_2_O, 10 μg/ml of Nab228 monoclonal antibody (Sigma-Aldrich, St. Louis, USA) in PBS was added to the wells and incubated for 1 h. After washing three times with TBS + 0.1%Tween20 (TBST) and TBS, each of the wells was blocked with Smartblock solution (Candor Bioscience, Germany) over night. The next day, samples and standards were added to the plate and incubated for 1 h. All samples were diluted tenfold in TBS. Aβ_(1–42)_-SiNaPs (silica nanoparticles) with a diameter of 20 nm and approx. 30 epitopes (Aβ_(1–42)_), synthesized and analyzed using the methods described previously [30], served as a calibration standard for Aβ oligomers. After washing the excessive sample away three times with TBS, 1.25 μg/ml mAb IC16 (epitope Aβ_1–8_) [40], labeled with CF-633 dye (Sigma-Aldrich, Germany), and 1.25 μg/ml Nab228 (epitope Aβ_1–11_), labeled with CF-488 dye (both: Sigma-Aldrich, Germany), both ultracentrifuged (100,000*g*, 1 h, 4 °C), were added to the wells and incubated for 1 h. After incubation, the excessive detection antibodies were washed away three times with TBS and the plate was sealed with a plastic foil and transferred to a Leica multi-color TIRF (total internal reflection fluorescence) system (AM TIRF MC, Leica Microsystems, Wetzlar, Germany). The TIRF system operated with an automated stage and a ×100 oil immersion objective (1.47 oil CORR TIRF Leica). Images were recorded consecutively with Ex/Em = 633/705 and 488/525 nm with a 500-ms exposure time and a gain of 800 for both color channels at a penetration depth of 200 nm. The microscope took 5 × 5 images per well in each channel, which corresponds to ca. 3% of the well’s surface. Each image consisted of 1000 × 1000 pixels with a lateral resolution of 116 nm (pixel to pixel) and an intensity resolution of 14 bit (gray scale).

Image analysis was performed using sFIDAta, a custom-made software. After removing “out of focus” images (ca. 5%), cutoff values were calculated for each channel based on the blank. The software then applied the cutoff values to the sample results and counted the pixels that were in both channels and at the same position, higher than the cutoff. The number of these co-localized pixels was taken as the sFIDA readout. Using the Aβ_(1–42)_-SiNaP standards, the sFIDA readout was converted to oligomer concentration. All samples were measured in triplicate.

For all sFIDA experiments, with the expectation the one performed with the three fractions also used for ELISA, 150 μl of brain homogenate was centrifuged at 1200*g* for 10 min. One hundred microliters of supernatant of each homogenate was loaded on the top of a density gradient consisting of 5 to 50% (*w*/*v*) iodixanol (OptiPrep, Axis-Shield, Norway). After centrifugation (3 h, 259,000×*g*, at 4 °C) (Optima TL-100, Beckman Coulter, USA), 14 fractions (140 μl each) were removed from top to bottom of the tubes. Ten microliters of each fraction was diluted in 90 μl TBS before being applied to the sFIDA microplates.

### Clinical Chemistry

Tests for the quantitative determination of lactate dehydrogenase (LDH), aspartate aminotransferase (AST), alanine aminotransferase (ALT), and alkaline phosphatase (AP) were performed with plasma samples from RD2- or placebo-treated mice, or their non-transgenic littermates, using Roche automated clinical chemistry analyzers (cobas 8000 modular analyzer series, Roche, Basel, Switzerland) according to the manufacturer’s protocol.

### Cytokine Assay

Plasma samples from RD2- or placebo-treated mice, or their non-transgenic littermates were tested for interleukin-1 alpha (IL-1α), interleukin-10 (IL-10), interleukin-12p40 (IL-12p40), interleukin-13 (IL-13), interleukin-17 (IL-17), granulocyte colony-stimulating factor (G-CSF), interferon gamma (IFN-γ), monocyte chemoattractant protein-1 (MCP-1), macrophage inflammatory proteins alpha and beta (MIP-1α and MIP-1β), regulated on activation, normal T cell expressed and secreted (RANTES), and tumor necrosis factor alpha (TNF-α) (Bio-Plex Pro Mouse Cytokine 23-plex Assay, Bio-Rad, CA, USA). The assay was performed following the manufacturer’s protocol. Samples that were out of the detection limit were excluded from analysis.

### Statistical Analysis

All statistical calculations were performed using GraphPad Prism 5 (GraphPad Software, Inc., La Jolla, USA) or SigmaPlot Version 11 (Systat Software, Germany). All data are represented as mean ± SEM. The Gaussian distribution of all data was tested in the D’Agostino and Pearson omnibus normality test. Normally distributed data were analyzed in the one-way analysis of variance (ANOVA) with Tukey post hoc analysis (nesting behavior, probe trial MWM) or unpaired one-tailed *t* test (6E10 staining). Data, which were not normally distributed, were tested with the Kruskal-Wallis test with Dunn’s multiple comparison test (marble burying, open field test, cytokine assay, IHC, clinical chemistry). MWM was analyzed with the use of InVivoStat 2.5 (InVivoStat by Simon Bate and Robin Clarke, UK) [41] with the repeated measures (RM) parametric analysis and SigmaPlot. The escape latency to the platform was considered as not normally distributed and therefore analyzed by the Friedman repeated measures ANOVA on ranks. Distance moved and the percentage duration in the platform quadrant were analyzed by a repeated measures two-way ANOVA. As the fractions generated from each brain homogenate (Tris, DEA, and FA fraction, or DGC fractions 1 to 14) were considered to be related, results from ELISA and sFIDA measurements were analyzed with a two-way RM ANOVA. *p* values smaller than 0.05 were considered to indicate significant statistical differences in the tests.

## Results

### RD2 Treatment Resulted in Improved Cognition and Behavior of Treated Mice, Indistinguishable from Non-transgenic Mice

In the present study, three groups of mice were tested. Two groups consisted of 18-month-old, transgenic APP/PS1 mice. In the first group, 11 mice were orally treated with RD2. Oral treatment was carried out by giving a daily dose of roughly 200 mg/kg of RD2 in one jelly (treatment group). In the second group, ten APP/PS1 mice were given a jelly without RD2 (placebo group, ten mice). The third group, consisting of 11 non-transgenic littermates, was left untreated (wild-type group) and assured for the quality of the behavioral assessments. At the end of the 12-week treatment period, different behavioral experiments were conducted to evaluate the treatment effect. Thereafter, different immunohistochemical and biochemical experiments were performed to investigate the treatment effect on Aβ pathology, inflammation, and possible side effects.

**Fig. 1:**
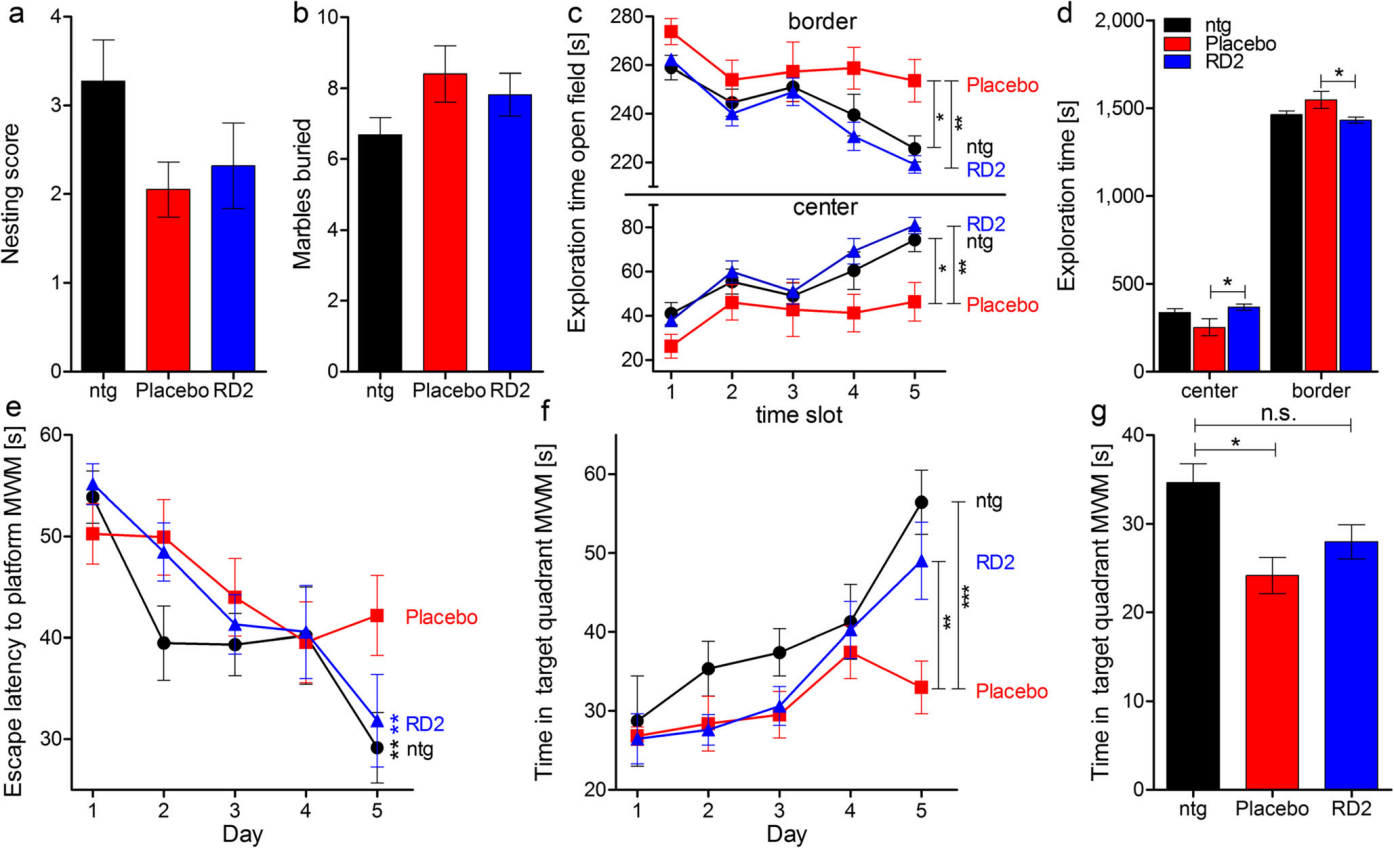
RD2 treatment of old-aged APP/PS1 mice resulted in significantly improved cognitive performance. Nesting behavior (**a**) and marble burying (**b**) were examined to investigate species-typical behavior. Neither RD2 nor placebo treatment had a significant influence on the nesting behavior, nor on the marble burying results, compared to non-transgenic littermates (ntg). An open field test was performed to analyze and compare the exploratory and anxiety-related behaviors of RD2- and placebo-treated mice, as well as their ntg (**c**, **d**). Mice were allowed to explore the arena, divided into border and center zones, for 25 min. Exploration of the center and border zones is represented as five different time slots (**c**). In contrast to placebo-treated mice, RD2-treated mice and ntg exhibited a habituation effect to the arena that significantly differs from the performance of placebo-treated mice (**c**). Analysis of overall exploration revealed a significant difference in the exploratory and anxiety-related behaviors between RD2- and placebo-treated mice but not between RD2-treated mice and ntg, indicating that RD2 treatment reversed the phenotype of the transgenic mice (**d**). Additionally, a Morris water maze (MWM) was conducted in which mice were trained for 5 days to find a hidden platform (**e**, **f**). Escape latencies to the platform of RD2-treated mice were significantly lower compared to placebo-treated mice, indicating improved learning, similar to ntg (**e**). In addition, RD2-treated mice and ntg spent significantly more time in the platform quadrant compared to placebo-treated mice on the last training day (**f**). Analysis of the probe trial revealed a significant difference between ntg and placebo-treated mice, but not between ntg and RD2-treated mice, respectively (**g**). Each behavioral performance of RD2-treated mice was similar to those of ntg, suggesting a reversed phenotype. Data is represented as mean ± SEM, RD2 *n* = 11, placebo *n* = 10, ntg *n* = 11

To assess the potential consequences of long-term oral treatment with RD2 over 12 weeks on species-typical behavior, and to investigate possible adverse side effects caused by the treatment, nesting behavior and marble burying, as basic behavioral tests, were performed with RD2- in comparison to placebo-treated APP/PS1 mice and their non-transgenic littermates (Fig. 1a, b). Neither nesting behavior nor marble burying revealed any significant differences between the three groups (Fig. 1a, b; nesting behavior score (mean ± SEM): ntg 3.3 ± 0.5, placebo 2.1 ± 0.3, RD2 2.3 ± 0.5, one-way ANOVA *F*_(2,31)_ = 2.21, n.s. *p* = 0.13; marble burying (mean ± SEM): ntg 6.6 ± 0.5, placebo 8.4 ± 0.8, RD2 7.8 ± 0.6, one-way ANOVA, *F*_(2,31)_ = 1.91, n.s. *p* = 0.17). Afterwards, an open field test was performed to assay general differences in exploratory and anxiety-related behaviors of the treated mice and their non-transgenic littermates. Analysis of the open field test did not reveal significant differences in the analyzed parameters between RD2-treated mice and non-transgenic littermates, but did between RD2- and placebo-treated mice. This indicates that phenotypic impairments of the transgenic mice were reversed by RD2 treatment (Fig. 1d; two-way ANOVA, *F*_(2,58)_ = 7.47, *p* = 0.001, Tukey post hoc analysis: ntg vs. placebo n.s. *p* = 0.14, ntg vs. RD2 n.s. *p* = 0.75, placebo vs. RD2 *p* = 0.03). In detail, RD2-treated mice stayed significantly longer in the center zone than placebo-treated mice (Fig. 1d), while the time spent in border and center zone did not significantly differ between RD2-treated mice and their non-transgenic littermates (Fig. 1d). This suggests decreased anxiety-related behavior of RD2- compared to the placebo-treated mice, again leading to reversal of the behavior of RD2-treated mice to the level of the non-transgenic littermates. Furthermore, there was also a difference in exploratory behavior during the course of the test. RD2-treated mice and non-transgenic littermates explored the center zone for significantly longer than the placebo-treated mice. Thus, RD2-treated mice and non-transgenic littermates showed the typical habituation effect to the new and so far unexplored arena, while this habituation effect was not present in placebo-treated transgenic mice (Fig. 1c; two-way RM ANOVA, *F*_(2,116)_ = 4.12, *p* = 0.03, Tukey post hoc analysis time slot 5: ntg vs. placebo *p* = 0.01, ntg vs. RD2 n.s. *p* = 0.76, placebo vs. RD2 *p* = 0.001). The presence of the habituation effect, or its absence, became especially prominent during the second half of the test, suggesting that RD2 treatment led to the reversal of the transgenic mouse phenotype towards the phenotype of the non-transgenic littermates.

The Morris water maze (MWM) was conducted for the assessment of cognitive impairment, especially for deficits in cognitive abilities, and in spatial learning and memory. During the 5-day training phase of the MWM, placebo-treated transgenic mice showed impaired performance without an apparent learning effect over time. In contrast, RD2-treated mice showed a significant learning effect over time in the training phase of the MWM, with their performance being indistinguishable from non-transgenic littermates (Fig. 1e; Friedman repeated measures (RM) ANOVA on ranks, RD2-treated mice *p* < 0.001, non-transgenic littermates *p* = 0.008, placebo-treated mice n.s. *p* = 0.1). Moreover, RD2-treated mice spent significantly more time in the target quadrant than the placebo-treated mice. Here, RD2-treated transgenic mice were also indistinguishable from non-transgenic littermates (Fig. 1f; two-way RM ANOVA, *F*_(2,116)_ = 4.39, *p* = 0.02, Tukey post hoc analysis day 5, ntg vs. placebo *p* < 0.001, ntg vs. RD2 n.s. *p* = 0.3, placebo vs. RD2 n.s. *p* = 0.006). Memory retrieval during the probe trial on day 6 did not reveal significant differences in performance between RD2-treated and placebo-treated mice, but did reveal differences between non-transgenic littermates and placebo-treated mice. The placebo-treated mice spent significantly less time in the quadrant, where the platform was located, than the non-transgenic littermates. This emphasizes the cognitive impairment of the placebo-treated transgenic mice (Fig. 1g; one-way ANOVA *F*_(2,31)_ = 6.77, *p* = 0.004, Tukey post hoc analysis, non-transgenic littermates vs. placebo *p* = 0.003, non-transgenic littermates vs. RD2 n.s. *p* = 0.06, placebo vs. RD2 n.s. *p* = 0.4).

### Treatment with RD2 Led to Significant Decrease of Aβ Plaque Load

**Fig. 2:**
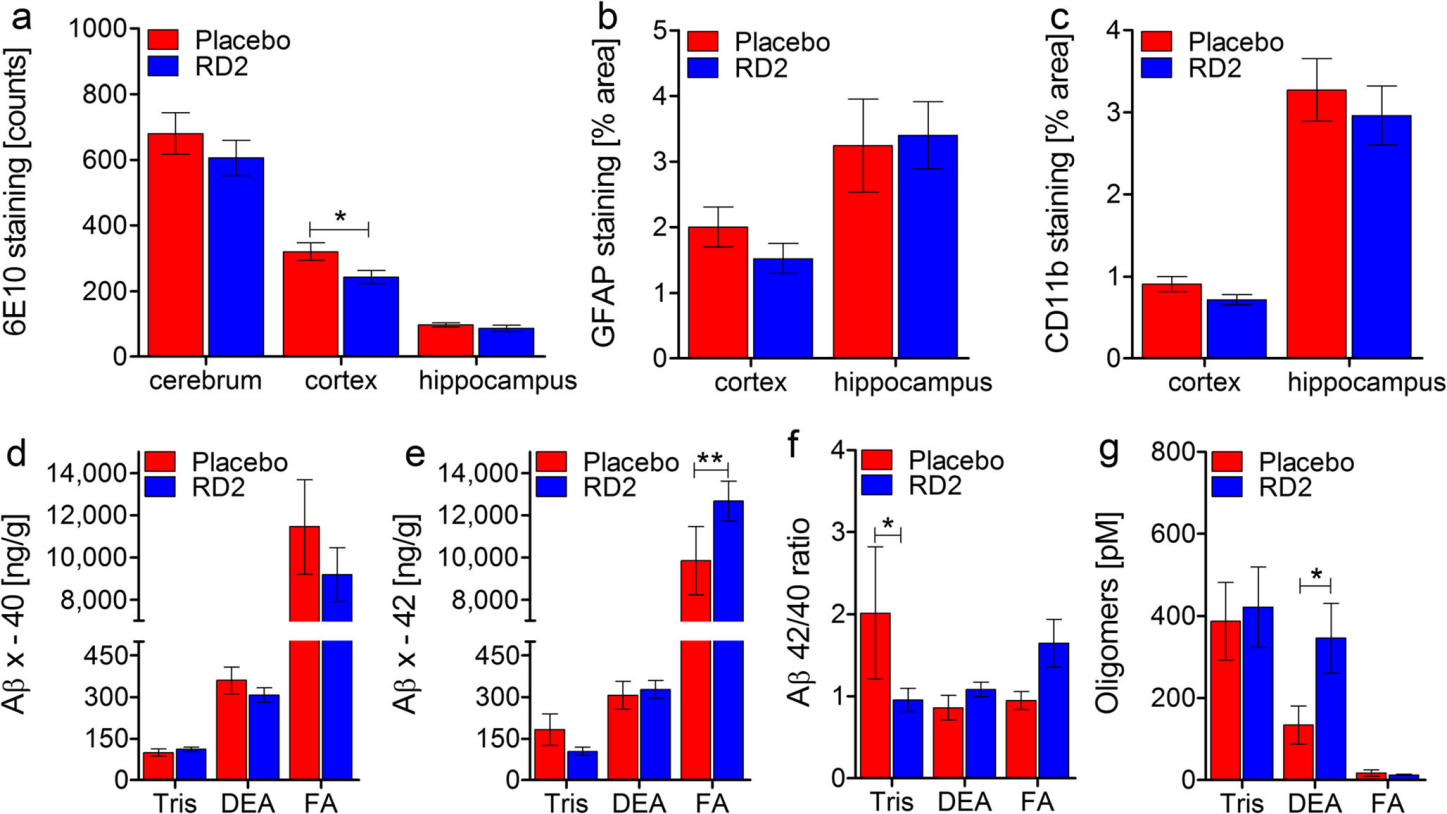
Effects of RD2 treatment on Aβ pathology, gliosis, and Aβ oligomers in the brains of APP/PS1 mice. Investigations of a potential reduction in either Aβ plaque load or astrogliosis after RD2 treatment were performed using immunohistochemical analysis. Plaque count was analyzed by 6E10 staining in different areas of the brain (cerebrum, cortex, and hippocampus). Compared to placebo-treated mice, RD2-treated mice showed a decreased number of Aβ deposits in all analyzed regions being significant in the cortex (**a**). Activated astrocytes were quantified after GFAP staining (**b**) and activated microglia were quantified after CD11b staining (**c**). Results exhibited no significant difference in gliosis between RD2- and placebo-treated mice. Levels of Aβ x–40 (**d**) and Aβ x–42 (**e**) in the Tris-soluble (Tris), DEA-soluble (DEA), and formic acid fractions (FA) of brain homogenates of RD2- and placebo-treated mice were analyzed by ELISA, resulting in a significant increase of Aβ(x-42) in the FA fraction of RD2-treated mice. Concentrations were presented in ng (Aβ(x–40) or Aβ(x–42))/g (brain). Aβ 42/40 ratio is shown in **f** and yielded a significantly higher ratio in the Tris fraction of placebo-treated mice. Aβ oligomer concentrations were analyzed in the abovementioned fractions by sFIDA assay, resulting in a significant increase in Aβ oligomers within the DEA fraction of RD2- compared to placebo-treated mice (**g**). Data is represented as mean ± SEM. All: * *p* > 0.05

The staining of brain tissue sections against human Aβ (6E10), activated astrocytes (GFAP), and activated microglia (CD11b) was accomplished to investigate the effects of long-term oral RD2 treatment on Aβ pathology and gliosis of the APP/PS1 transgenic mouse model [33, 34]. Figure 2a–c shows the results of 6E10, GFAP, and CD11b staining with subsequent quantification of different brain areas (cerebrum, cortex, hippocampus). Overall, the data suggest that RD2 treatment led to a reduction of Aβ plaque load (Fig. 2a). This became significant only for the Aβ plaque load in the cortex (unpaired one-tailed Student’s *t* test, *p* = 0.02). There was no significance for the Aβ plaque load reduction of the whole cerebrum or the hippocampus (unpaired one-tailed Student’s *t* test, cerebrum n.s. *p* = 0.2, hippocampus n.s. *p* = 0.1). We observed a tendency for a reduction in gliosis after RD2 treatment, which was close to being significant in the cortex of RD2-treated mice (Fig. 2b; GFAP staining: unpaired one-tailed Student’s *t* test, cortex n.s. *p* = 0.064, hippocampus n.s. *p* = 0.3) (Fig. 2c; CD11b staining: unpaired one-tailed Student’s *t* test, cortex n.s. *p* = 0.051, hippocampus n.s. *p* = 0.28).

Biochemical quantitation of Aβ(x–40) and Aβ(x–42) was accomplished with three fractions (Tris-soluble, DEA-soluble, and formic acid (FA) fractions) prepared from brain homogenates of RD2-treated and placebo-treated mice (Fig. 2d–g). There were no significant differences for the contents of Aβ(x–40) in any of the fractions from RD2-treated mice compared to placebo-treated mice (Fig. 2d; two-way RM ANOVA treatment, *F*_(1,32)_ = 0.87 n.s., *p* = 0.365; fractions, *F*_(2,32)_ = 69.19, *p* < 0.001; interaction, *F*_(2,32)_ = 0.85, n.s., *p* = 0.44; Tukey post hoc analysis RD2 vs. placebo, Tris fraction n.s. *p* = 0.99, DEA fraction n.s. *p* = 0.97, FA fraction n.s. *p* = 0.12). RD2-treated mice showed a significant increase of Aβ(x–42) in the FA fraction (Fig. 2e; two-way RM ANOVA treatment, *F*_(1,32)_ = 2.32 n.s., *p* = 0.15; fractions, *F*_(2,32)_ = 155.58, *p* < 0.001; interaction, *F*_(2,32)_ = 2.61, n.s., *p* = 0.089; Tukey post hoc analysis RD2 vs. placebo, Tris fraction n.s. *p* = 0.94, DEA fraction n.s. *p* = 0.98, FA fraction *p* = 0.009). The Aβ42/40 ratio was nearly 1 in all samples, with the exception of the Tris fraction of placebo-treated mice, which differed significantly from the Tris fraction of RD2-treated mice (Fig. 2f; two-way RM ANOVA treatment, *F*_(1,32)_ = 0.026 n.s., *p* = 0.88; fractions, *F*_(2,32)_ = 1.26 n.s., *p* = 0.3; interaction, *F*_(2,32)_ = 3.85, n.s., *p* = 0.032; Tukey post hoc analysis RD2 vs. placebo, Tris fraction *p* = 0.03, DEA fraction n.s. *p* = 0.64, FA fraction n.s. *p* = 0.146). Analysis of Aβ oligomer concentration within the Tris, DEA, and FA fraction was conducted using the surface-based fluorescence intensity distribution analysis (sFIDA) assay. Results displayed a significant increase in Aβ oligomers within the DEA fraction of RD2-treated mice, compared to placebo-treated mice (Fig. 2g; two-way RM ANOVA treatment, *F*_(1,16)_ = 1.48 n.s., *p* = 0.24; fractions, *F*_(1,16)_ = 5.68, *p* = 0.03; interaction, *F*_(2,32)_ = 1.66, n.s., *p* = 0.22; Tukey post hoc analysis RD2 vs. placebo, Tris fraction n.s. *p* = 0.73, DEA fraction *p* = 0.04, FA n.s. *p* = 0.97). The sFIDA assay is highly specific and sensitive to aggregated Aβ species and completely insensitive to monomers. It was assumed that all protein assemblies from the FA-solubilized pellet were fully denatured and dissolved. Indeed, the FA fractions yielded Aβ oligomer concentrations close to zero.

### Measurement of Aβ Oligomer Concentrations by the sFIDA Assay Revealed a Significant Reduction of Aβ Oligomers in RD2-Treated Mice

**Fig. 3:**
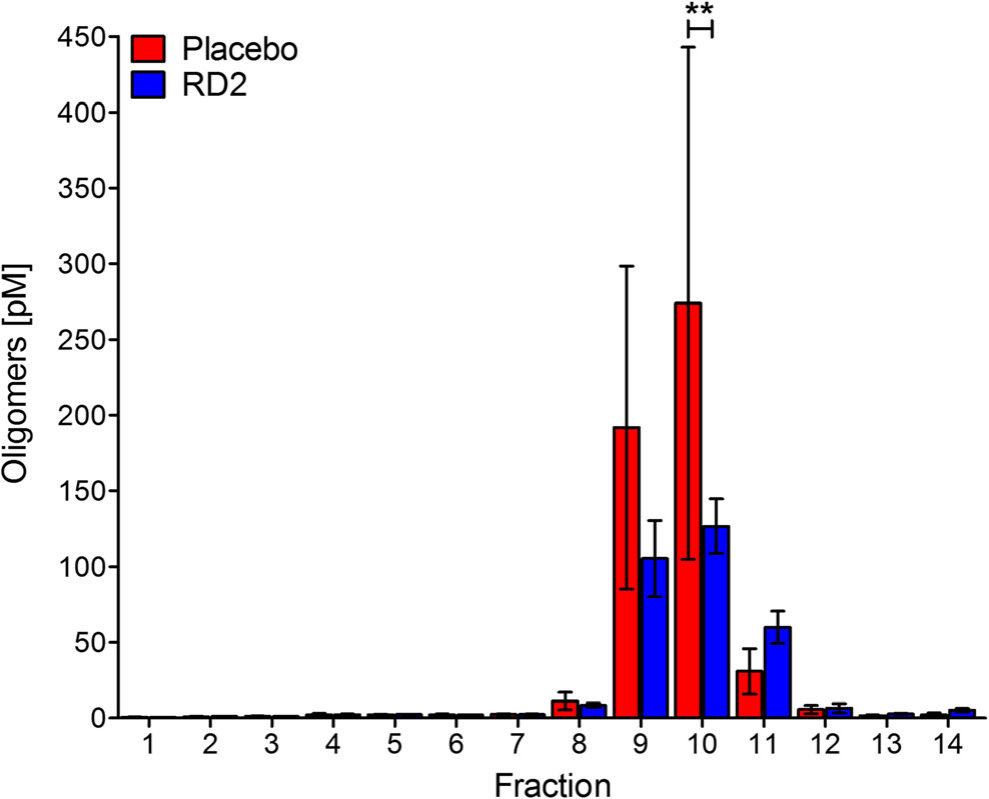
Treatment with RD2 significantly reduced oligomeric Aβ species. Oligomeric Aβ levels in the fractions of density gradient ultracentrifuged brain homogenates of RD2- and placebo-treated APP/PS1 mice were analyzed by the aggregate-specific and highly sensitive sFIDA assay. Results revealed a significant decrease in oligomeric Aβ species in fraction 10 of RD2- compared to placebo-treated mice. sFIDA readout was converted to Aβ oligomer concentrations after calibration with Aβ_1–42_-SiNaP (internal standard calibration) (silica nanoparticles). Data is represented as mean ± SEM. Placebo *n* = 4, RD2 *n* = 5. **p < 0.01

RD2 was designed to directly and specifically eliminate Aβ oligomers. To develop a suitable method to investigate target engagement in vivo, an assay able to quantitate Aβ oligomers in body liquids (e.g., cerebrospinal fluid, plasma) was developed. The assay, called sFIDA, is insensitive to Aβ monomers and achieves single-particle sensitivity [26, 29, 30]. In the present study, we developed this technique further in order to measure Aβ oligomers in organ homogenates. Therefore, we fractionated full brain homogenates of RD2- and placebo-treated mice by density gradient centrifugation (DGC) according to particle sizes and applied each of the 14 obtained fractions to the sFIDA assay. The sFIDA assay combines the specificity of immunological assays with the sensitivity of high-resolution fluorescence microscopy, which allows a lower limit of detection, down to the single aggregate level. The selectivity for aggregated species is realized by using anti-Aβ antibodies for capturing and probing, which recognize overlapping epitopes located at the N-terminus of Aβ subunits [27, 40]. To test for successful target engagement due to RD2 treatment, we compared DGC-fractionated brain homogenates of RD2- and placebo-treated mice. The result is shown in Fig. 3 and reveals a significant decrease of Aβ oligomer levels of RD2- compared to placebo-treated mice, especially in fraction 10 (Fig. 3e; two-way RM ANOVA treatment, *F*_(1,91)_ = 0.69 n.s., *p* = 0.44; fractions, *F*_(13,91)_ = 7.3, *p* < 0.001; interaction, *F*_(13,91)_ = 0.96, n.s., *p* = 0.49; Tukey post hoc analysis RD2 vs. placebo, Tukey post hoc analysis fraction 10 *p* = 0.003, fractions 1 to 9 and 11 to 14: n.s.).

### Treatment with RD2 Did Not Affect Plasma Levels of Different Cytokines

The plasma cytokine levels of RD2- and placebo-treated mice, and their non-transgenic littermates were determined using the Bio-Plex Pro Mouse Cytokine 23-plex assay (Table 1). Some values were below the limit of detection (LoD) and were therefore excluded from evaluation. The only significant difference found between RD2- and placebo-treated mice was the IL-1α level (Table 1, Kruskal-Wallis one-way ANOVA on ranks *p* = 0.04, ntg vs. placebo n.s., ntg vs. RD2 n.s., placebo vs. RD2 *p* < 0.05). There was no significant difference between RD2-treated mice and non-transgenic littermates. Additionally, a significant reduction in MIP-1α in placebo-treated mice compared to non-transgenic littermates was observed, but not between RD2- and placebo-treated mice (Table '1, Kruskal-Wallis one-way ANOVA on ranks *p* = 0.008, ntg vs. placebo *p* < 0.05, ntg vs. RD2 n.s., placebo vs. RD2 n.s.).

**Table 1:** Cytokine assay of non-transgenic littermates, RD2- and placebo-treated mice

[pg/ml]	ntg	Placebo	RD2	Statistic
IL-1α	23.54 ± 4.89	24.42 ± 2.16*	15.1 ± 2.18*	Placebo vs. ntg n.s. RD2 vs. placebo *p* < 0.05 RD2 vs. ntg n.s.
IL-10	22.17 ± 5.76	39.44 ± 18.52	26.89 ± 6.78	n.s.
IL-12 (p40)	191.7 ± 19.39	187.0 ± 24.30	208.9 ± 18.22	n.s.
IL-13	147.9 ± 26.83	106.8 ± 32.31	176.2 ± 44.57	n.s.
IL-17	2.25 ± 0.42	5.03 ± 1.53	5.85 ± 1.80	n.s.
G-CSF	187.6 ± 61.63	116.2 ± 29.08	118.2 ± 19.14	n.s.
IFN-γ	16.67 ± 3.46	11.09 ± 2.19	18.38 ± 4.89	n.s.
MCP-1	131.2 ± 19.31	156.4 ± 26.47	146.0 ± 27.46	n.s.
MIP-1α	31.97 ± 3.31*	14.75 ± 2.23*	28.03 ± 6.02	Placebo vs. ntg *p* < 0.05 RD2 vs. placebo n.s. RD2 vs. ntg n.s.
MIP-1β	19.72 ± 3.99	22.23 ± 4.15	30.27 ± 6.93	n.s.
RANTES	27.15 ± 10.4	16.55 ± 4.32	25.88 ± 9.57	n.s.
TNF-α	64.12 ± 7.32	87.89 ± 14.02	106.6 ± 28.51	n.s.

For the evaluation of a potential change in the amount of different cytokines, a Bio-Plex Pro Mouse Cytokine 23-plex Assay was performed with heparinized plasma samples of non-transgenic littermates (ntg), RD2- and placebo-treated mice. Data revealed a significant reduction in IL-1α due to RD2 treatment in comparison to placebo treatment. Furthermore, significantly decreased concentrations of MIP-1α were detected in placebo-treated mice compared to ntg, but no differences between RD2-treated mice and ntg were observed. Cytokine concentrations are given as picograms per milliliter. Data is represented as mean ± SEM, * < 0.05, *n.s.* not significant

### Adverse Drug Reactions Have Not Been Observed After Long-term Oral Treatment with RD2

**Fig. 4:**
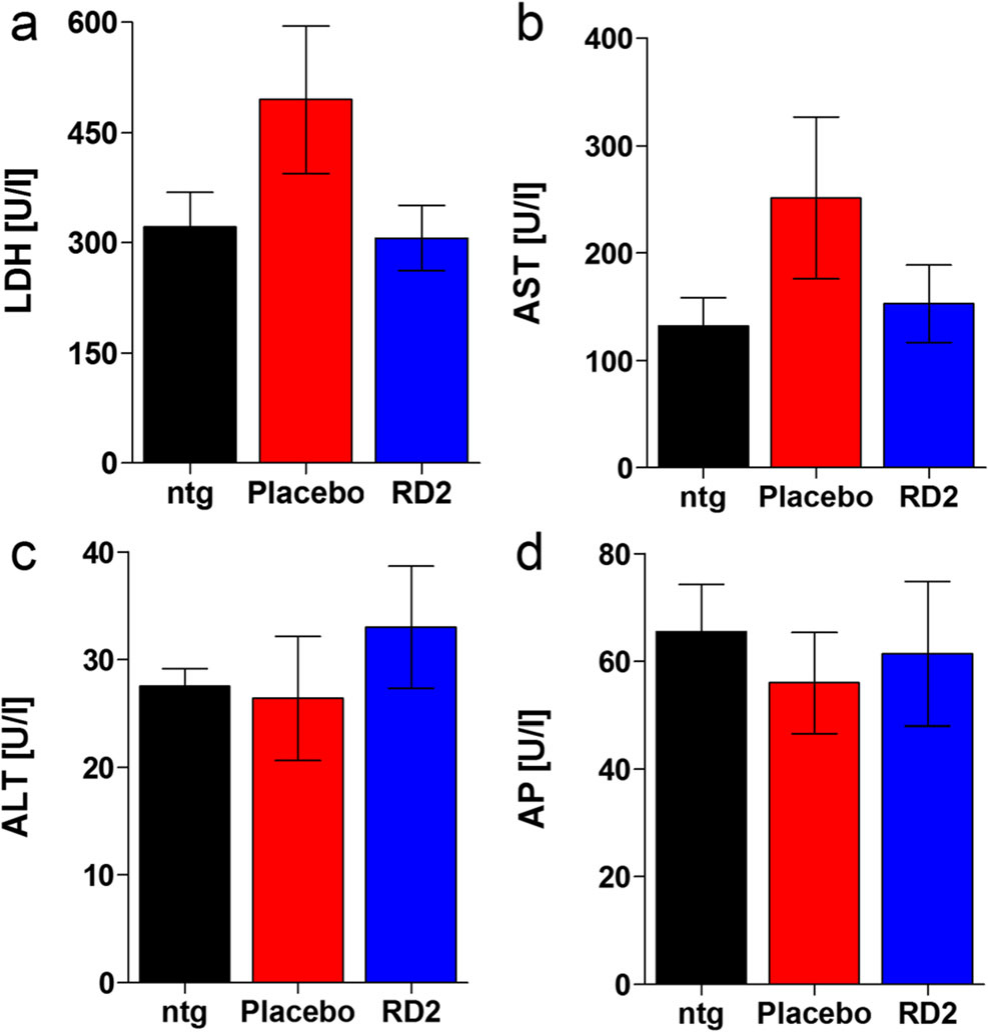
Effects of long-term RD2 treatment on different plasma enzymes. Examination of lactate dehydrogenase (LDH) (**a**), aspartate aminotransferase (AST) (**b**), alanine aminotransferase (ALT) (**c**), and alkaline phosphatases (AP) (**d**). These plasma enzymes, usually analyzed to investigate whether a treatment with a new medication induces, e.g., liver intoxication, indicated no toxic adverse side effects due to long-term RD2 treatment. No significant differences were detected between non-transgenic littermates (ntg), RD2- or placebo-treated APP/PS1 mice. This is particularly important based on the high concentration administered (200 mg/kg daily). Data is represented as mean ± SEM. Kruskal-Wallis one-way ANOVA on ranks, (**a**): n.s. *p* = 0.33; one-way ANOVA, (**b**): *F*(_2,13_) = 1.42 n.s. *p* = 0.28, (**c**): *F*(_2,13_) = 0.5 n.s. *p* = 0.62, (**d**): *F*(_2,13_) = 0.18 n.s. *p* = 0.84. ntg *n* = 4, placebo *n* = 5, RD2 *n* = 5

To test for any adverse or even toxic side effects of the RD2 treatment, four plasma parameters were analyzed: lactate dehydrogenase (LDH), aspartate aminotransferase (AST), alanine aminotransferase (ALT), and alkaline phosphatase (AP). These parameters are also analyzed in clinical routine to give indications about possible drug-mediated liver or heart-targeted toxicity. The results did not reveal any changes in these parameters (Fig. 4a–d). Additionally, RD2 treatment did not result in significant gain or loss of body weight (before vs. after treatment, mean ± SEM: RD2: 34.4 ± 1.0 vs. 33.5 ± 0.9 g, placebo: 34.3 ± 0.9 vs. 34.2 ± 0.7 g, non-transgenic littermates 36.4 ± 1.1 vs. 34.1 ± 0.8 g) or a change in the general physiological or behavioral condition of the mice.

## Discussion

In the present study, we examined the drug candidate RD2 for true therapeutic, rather than only preventive efficacy, in old-aged transgenic APP/PS1 mice. RD2 has previously been proven to specifically eliminate toxic Aβ oligomers using the Aβ-QIAD assay and to reduce the formation of Aβ_(1–42)_ fibrils and their seeding potential in vitro [22]*.* In vivo, it could be shown that RD2 has very favorable pharmacokinetic properties [42]. Additionally, the intraperitoneal administration of RD2 over 4 weeks led to significant cognitive improvement in young APP/PS1 mice that displayed little Aβ pathology at the beginning of the treatment. This study clearly demonstrates that RD2 is able to block progression of the disease. During a second study using the APP Swedish London mouse model, it was possible to prove that oral treatment with RD2 also leads to a significant improvement in cognition and memory. Here, we decided to challenge the efficiency of RD2 by orally treating old-aged APP/PS1 mice with full-blown pathology over 12 weeks, as this may mimic the patients’ situation more closely at moderate and more advanced disease stages, in respect to plaque pathology and cognitive deficits.

As a result of the treatment, we were able to demonstrate the curative in vivo efficacy of RD2. It is very well documented that APP/PS1 mice develop cognitive deficits by 7 months of age, which are clearly pronounced at the age of 18 months [43–45]. Because the cognitive abilities of RD2-treated mice were significantly improved compared to the placebo-treated mice, and were indistinguishable from non-transgenic littermates, we conclude that RD2 treatment led to an overall reversal of the cognitive impairments in the transgenic mice. This is supported by the observations made in the open field test, where the behavior of RD2-treated mice was indistinguishable from non-transgenic littermates.

RD2 treatment over 12 weeks decreased the Aβ plaque load, which became significant in the cortex. Such a significant reduction in Aβ plaque load has not been observed in previous RD2 treatment studies, which had shorter treatment durations and used lower RD2 doses, but nevertheless yielded significant improvement in cognition [22, 23]. We conclude that cognitive improvement is not dependent on Aβ plaque load reduction. This is in line with the well-known fact that plaque load does not correlate with cognitive decline in humans [46]. The observation that cognitive improvement is achieved with shorter treatment duration and lower doses of RD2 before any reduction of plaque load becomes significant might have implications for future clinical study designs. Therefore, one may carefully consider whether plaque load should be a primary efficacy endpoint in clinical studies for the treatment of AD. As we had not observed significant plaque load reductions in the previously reported RD2 treatment studies with shorter treatment duration and lower doses, we were not surprised that there had not been a significant reduction in activated astrocytes and microglia in those studies. The significant reduction in cortex plaque load in the present study also led to decreased gliosis, although not to a significant extent. Due to the manifested phenotype of the utilized mouse model, it is likely that the chronic inflammation could not be fully reversed during the course of the experiment and that an even longer treatment period would have been necessary to act significantly on cerebral inflammation.

RD2 was designed to specifically and directly eliminate toxic Aβ oligomers. Such target engagement has already been shown in vitro using the QIAD assay [22], where RD2 had reduced the most toxic Aβ oligomers in DGC fractions 4 to 6 very significantly, resembling particle sizes of about 100 kDa [14]. To develop an experiment that allows the investigation of target engagement in vivo, we decided to use brain homogenates without enrichment steps for human Aβ, as this could potentially lead to the destruction of native Aβ oligomers and also to the formation of artificial Aβ aggregates formed during Aβ precipitation steps. Because the brain homogenates contain not only Aβ, but also all other brain-derived components, we used the ultra-sensitive and specific sFIDA assay [26, 29–31] for the quantification of Aβ oligomers in the DGC-fractionated brain homogenates ex vivo. The most significant reduction in Aβ containing particles by RD2 treatment was observed in fraction 10, which corresponds to particles with a molecular weight of larger than 400 kDa [14]. We speculate that the elevated molecular weight of those ex vivo obtained particles, as compared to the in vitro generated Aβ oligomers of the Aβ-QIAD [14], was due to other proteins, besides Aβ, that can be expected to be attached to Aβ oligomers. To the best of our knowledge, this is the first time that a reduction of Aβ oligomers has been shown in vivo and confirms the proposed mechanism of action for RD2 to be valid also in the functional brain.

Based on the results of additional assays probing for species-typical behavior, like marble burying and nesting behavior [36, 37, 47, 48], we conclude that RD2 treatment did not cause any adverse side effects affecting behavior. Moreover, the lack of significant changes in plasma concentrations of enzymes such as AST, ALT, LDH, and AP between RD2- and placebo-treated mice, as well as their non-transgenic littermates, further supports the absence of any adverse side effects. Otherwise, changes in plasma concentrations of these enzymes would have given indications about liver- or heart-targeted toxicity [49]. This is particularly important, because relatively high doses of RD2 were administered daily over 12 weeks.

In contrast to active and passive immunization against Aβ species, the postulated mechanism of action of RD2 does not require components of the immune system. Therefore, one would not expect a significant activation of the immune system, which would be indicated, for example, by significant changes in plasma inflammation markers. Indeed, determination of various inflammation markers did not yield any signs of immune system activation upon RD2 treatment. The necessity of having to rely on the immune system might lead to a negative activation of, e.g., T cells, in the worst case resulting in adverse side effects (e.g., microhemorrhages or meningoencephalitis) [50]. These adverse side effects were visible occasionally during the clinical testings of the first generation of Aβ immunization, e.g., bapineuzumab and solanezumab [51, 52]. A further advantage of RD2, in comparison to anti-Aβ antibodies, is the oral availability. Pharmacokinetic profiles of RD2 and its lead compound D3 revealed high oral bioavailabilities [20, 42]. Oral administration is the most attractive application method in humans and usually leads to the highest possible compliance.

## Conclusion

Here, we describe the effects of a truly curative, oral, long-term treatment of old-aged APP/PS1 mice with full-blown pathology. We were able to demonstrate that RD2 reversed the cognitive and behavioral deficits in these old transgenic mice to the levels of non-transgenic littermates. Moreover, we demonstrated in vivo target engagement of RD2 on oligomeric Aβ species.

Even though the mice were treated with a relatively high dose, this was tolerated very well and no obvious adverse drug effects were observed. This strengthens the hypothesis that the observed improvement in cognition was due to the direct and specific reduction of oligomers, further supporting Aβ oligomer elimination as a successful therapeutic strategy against Alzheimer’s disease.
